# Occupational skin health risk among nurses: development and validation of a nomogram-based prediction model

**DOI:** 10.3389/fpubh.2026.1748717

**Published:** 2026-03-31

**Authors:** Yi Xu, Ting Lu, Lixiang Feng, Yingying Chen, Siyan Chen, Rongsheng Xiong

**Affiliations:** 1 Department of Thoracic Surgery, Nanxishan Hospital of Guangxi Zhuang Autonomous Region (The Second People’s Hospital of Guangxi Zhuang Autonomous Region), Guilin, Guangxi, China; 2 Department of Community Health, Cancer Research and Specialist Centre, School of Medical Sciences, Universiti Sains Malaysia (USM), George Town, Penang, Malaysia; 3 Day Chemotherapy Center, The First Affiliated Hospital of Guangxi Medical University, Nanning, Guangxi, China; 4 Guangxi Agricultural Engineering Vocational Technical College, Chongzuo, Guangxi, China; 5 Department of Oncology, The First Affiliated Hospital of Guangxi Medical University, Nanning, Guangxi, China

**Keywords:** calibration, decision curve analysis, logistic regression, nomogram, nurses, occupational contact dermatitis, occupational health, public health

## Abstract

**Background:**

Occupational contact dermatitis (OCD) is a prevalent work-related skin condition among nurses and remains a significant occupational health issue due to its impact on well-being, productivity, and workforce sustainability. However, reliable tools for early risk stratification in this population are lacking. This study aimed to develop and validate a nomogram-based prediction model to estimate the individual risk of OCD among nurses.

**Methods:**

A multicenter cross-sectional survey was conducted among 2,852 nurses from 40 hospitals across China. Participants were randomly assigned to a training cohort (*n* = 2,000) and a validation cohort (*n* = 852). Independent predictors were identified using univariate and multivariable logistic regression analyses. A nomogram was constructed based on the final multivariable model. Model performance was assessed using the area under the ROC curve (AUC), bootstrapped calibration plots, and decision curve analysis (DCA).

**Results:**

Nine predictors were independently associated with OCD: age, dermatitis history, glove type, glove-wearing hours, handwashing frequency during work, hospital level, hand-cream habit, baseline skin condition, and sleep duration. The model showed excellent discrimination (AUC = 0.925 in the training set; 0.931 in the validation set). Calibration curves demonstrated close agreement between predicted and observed risks. DCA indicated consistently higher net benefit compared with the “treat-all” and “treat-none” strategies across wide threshold probability ranges (0.01–0.98 in the training set; 0.02–0.96 in the validation set). The resulting nomogram provides an intuitive, point-based tool for individualized OCD risk prediction.

**Conclusion:**

A robust, well-validated prediction model and nomogram were developed to estimate OCD risk among nurses. This tool may support occupational health screening, early risk identification, and targeted preventive strategies in healthcare institutions.

## Introduction

Occupational contact dermatitis (OCD) is among the most prevalent work-related skin conditions globally and represents a persistent occupational and public health challenge in healthcare environments (1, 2). Clinically, contact dermatitis is an inflammatory skin disorder characterized by erythema, pruritus, scaling, vesiculation, or fissuring, most commonly affecting the hands. Epidemiological evidence indicates that occupational contact dermatitis represents a substantial disease burden among nurses. A systematic review reported that the incidence of occupational contact dermatitis among healthcare workers ranged from 0.6 to 6.7 per 10,000 person-years in registry-based studies and from 15.9 to 780.0 per 10,000 person-years in cohort studies, with particularly high incidence observed among nurses and apprentice nurses, highlighting their elevated occupational risk (3). These findings underscore the substantial occupational burden of hand dermatitis in nursing populations and justify the need for early risk stratification and individualized preventive strategies. Nurses are disproportionately affected due to their high-frequency exposure to wet work, repeated hand hygiene procedures, disinfectants, and extended glove use—all of which compromise the skin barrier and elevate the risk of irritant or allergic reactions (3, 4). Beyond cutaneous symptoms, OCD can impair job performance, increase absenteeism, reduce workforce retention, and contribute to psychological burden, thereby threatening the sustainability of healthcare delivery and patient safety (5).

Despite progress in occupational protection and infection control protocols, the burden of hand dermatitis among nurses remains substantial worldwide (6, 7). Frequent contact with alcohol-based hand rubs, chlorhexidine, and synthetic or latex gloves continues to trigger OCD, especially in individuals with a history of allergic or dermatologic conditions (8). However, OCD is frequently underreported and inadequately managed in clinical practice, allowing early-stage dermatitis to advance and diminishing the effectiveness of conventional preventive strategies (9). These limitations underscore the need for proactive, person-centered risk identification methods integrated within occupational health programs.

In recent years, predictive modeling has gained increasing traction in clinical epidemiology and public health for early risk assessment and individualized prevention (10, 11). Nomogram-based models offer an interpretable and clinically applicable tool to quantify multifactorial risk by integrating diverse individual and occupational exposures (12, 13). Nevertheless, such approaches remain underutilized in occupational skin disease research, particularly among healthcare workers. Generic occupational health screenings often rely on isolated exposure indicators and may fail to capture the complex interplay between organizational factors (such as hospital tier), behavioral factors (such as sleep duration), and cumulative chemical exposures that jointly shape OCD risk among nurses. Given the multifactorial etiology of OCD, a validated predictive model tailored to nurses could improve early identification of high-risk individuals, inform targeted intervention, and optimize the allocation of limited occupational health resources. Therefore, this study aimed to construct and validate a nomogram-based model for estimating individual OCD risk among nurses using multicenter data across China, with the goal of supporting early risk stratification and targeted preventive strategies in occupational health practice.

## Methods

### Study design

This study employed a multicenter cross-sectional design to construct and validate a clinical prediction model for OCD of the hands among nurses. The methodology adhered to key principles outlined in the TRIPOD (14) (Transparent Reporting of a multivariable prediction model for Individual Prognosis or Diagnosis) statement, including pre-specification of candidate predictors, standardized outcome assessment, and transparent reporting of model-building and validation procedures. All data were collected from nurses across 40 hospitals covering multiple provinces and regions in China, using a structured electronic questionnaire distributed through a unified platform. The dataset was anonymized before analysis to ensure the integrity and reproducibility of the prediction model. Both the training and validation cohorts were drawn from the same pool of 40 participating hospitals, with nurses randomly allocated into training and validation sets to enable internal split-sample validation. This internal validation strategy was pre-specified to evaluate model reproducibility and to reduce the risk of overfitting during model development, in accordance with TRIPOD recommendations.

### Ethical approval

This study was reviewed and approved by Guangxi Zhuang Autonomous Region Nanxishan Hospital Ethics Committee (Approval no: NXSYY-2024-189(Y)). Completion of the online questionnaire was deemed to imply informed consent, as approved by the ethics committee. Participation was entirely voluntary, and respondents could withdraw at any time prior to submitting the questionnaire. No identifiable personal information was collected at any stage, and all data were processed anonymously. The study adhered to the ethical principles of the Declaration of Helsinki and followed institutional and national guidelines for human subjects’ research.

### Study population

Registered nurses from participating hospitals were recruited through an online survey distributed via the Questionnaire Star® platform. Eligible participants were required to meet all of the following criteria: (1) age ≥18 years; (2) currently employed as a nurse at the time of study initiation; (3) working in a direct patient-care role, defined as spending ≥50% of working time on bedside clinical care (e.g., inpatient wards, ICUs, operating rooms, emergency departments, or infusion clinics); (4) tenure in the current position for ≥3 months to ensure sufficient occupational exposure; (5) at least one working shift in the preceding 2 weeks (to exclude prolonged exposure interruption); (6) completion of all core questionnaire items, including OCD outcome and major exposure variables; (7) provision of a unique valid response from a participating hospital; and (8) completion time ≥90 s and passing basic quality-control checks. Participants were excluded if they met any of the following conditions: (1) non-clinical roles, such as administrative, managerial, educational, research, IT, or logistics positions; (2) nursing students, trainees, or other non-employed personnel; (3) continuous leave ≥30 days prior to the survey; (4) duplicate or suspicious submissions identified through phone number, employee ID, or device fingerprint, for which only the earliest and most complete record was retained; (5) missing outcome data (OCD status) or >20% missing key predictors; or (6) failure of quality-control screening, including completion time <90 s, straight-lining response patterns, or internal logical inconsistencies (e.g., reporting “never performs hand hygiene” while selecting “alcohol-based hand rub >20 times/day”). A total of 2,852 nurses fulfilled the inclusion criteria and were retained for analysis.

### Data collection

Data were collected using a structured, self-administered electronic questionnaire delivered through the Questionnaire Star® platform between January and June 2024. The survey was conducted across multiple secondary and tertiary hospitals in several provinces of China, including Guangxi, Guangdong, Hunan, Hubei, Jiangsu, and Shandong, ensuring broad geographic and institutional representation. A standardized distribution procedure was used. The research team provided the survey link and QR code to head nurses in participating centers, who disseminated them to departmental WeChat groups. Nurses completed the questionnaire on mobile devices, and responses were automatically stored on the secure Questionnaire Star® server before being downloaded for data cleaning. Multiple quality control procedures were applied, including screening for insufficient completion time (<90 s), straight-line or patterned responses, logical inconsistencies, and potential duplicate entries based on device or respondent metadata. Records failing any QC criteria were removed prior to analysis.

### Outcome definition

The primary outcome was occupational contact dermatitis of the hands (OCD), coded as a binary variable (0 = no, 1 = yes). Participants were specifically asked whether they had experienced hand dermatitis within the past 12 months that had been clinically diagnosed or confirmed by a dermatologist or other qualified physician. Self-perceived skin symptoms or self-diagnosed conditions were not considered sufficient for outcome classification, in order to enhance diagnostic reliability in the absence of contemporaneous clinical examination. This ensured that the outcome reflected clinically validated OCD rather than self-diagnosis. All outcome data were collected before model development to minimize incorporation bias.

### Candidate predictors

Candidate predictors were pre-specified based on clinical relevance, literature evidence, and feasibility of measurement in routine nursing practice. Predictors were collected while nurses were unaware of their outcome classification to avoid information bias. Variables included: Sociodemographic and professional characteristics: gender, age, education level, job title, hospital level, years of experience, department. Medical and dermatological history: allergy history, dermatitis history. Hand hygiene–related factors: handwashing frequency at work, main hand hygiene method, disinfectant type, sanitizer brand, frequency of soap washing, frequency of sanitizer use. Glove use: glove type (nitrile, PE, PVC, latex), glove-wearing hours per day. Skin care behaviors: hand cream use frequency, hand care habits, hand-drying method. Baseline skin condition: normal, dry, rough, or erythematous appearance. Lifestyle factor: daily sleep duration. All predictors were treated as categorical variables using clinically meaningful groupings. Categorization thresholds were determined based on clinical relevance, prior occupational health literature, and the distribution of responses in the study population to enhance interpretability and practical applicability.

### Sample size considerations

A total of 2,852 nurses were included in the final analysis, of whom approximately 11% had OCD, yielding more than 300 events. This exceeded the recommended minimum of 10–20 events per variable, allowing the inclusion of up to nine predictors in the final model while controlling overfitting risk and maintaining model stability.

### Handling of missing data

Missing data were minimal (<3%) and were handled using *complete-case analysis* (listwise deletion) to maintain consistency with the analytic protocol and avoid assumptions unsupported by the data. Only participants with complete information for the outcome and all candidate predictors were included in model development. Given the very low proportion of missing data and the absence of evidence for systematic missingness, complete-case analysis was considered unlikely to introduce substantial bias or loss of statistical efficiency; therefore, multiple imputation was not pursued to avoid unnecessary model complexity.

### Data preprocessing and dataset splitting

Following data cleaning, the full dataset was randomly divided into a training set (70%) and a validation set (30%) using stratified sampling. The two subsets were subsequently recombined with a new indicator variable (“dataset”: 1 = training, 0 = validation) to support transparent reporting. All categorical variables were converted into labeled factors before modeling. Stratified random sampling was applied to preserve outcome prevalence between datasets, ensuring comparable case-mix distributions and enhancing the robustness of internal validation.

### Statistical analysis

All statistical analyses were performed using R software (version 4.5.1). Baseline characteristics of the training and validation sets were summarized using the compareGroups package and compared with chi-square tests. Before model development, all categorical predictors were recoded as factor variables with predefined levels. Univariable logistic regression analyses were conducted using the glm() function to screen candidate predictors (*p* < 0.05), followed by multivariable modeling using four pre-specified strategies: enter (full) model, forward selection, backward elimination, and bidirectional stepwise selection. Stepwise procedures were implemented with the step () function, and models were compared using Akaike Information Criterion (AIC) and likelihood ratio tests. Model discrimination was assessed using area under the ROC curve (AUC) computed by the pROC package. Calibration was evaluated using observed-versus-predicted plots via val. Prob.(), as well as 1,000-bootstrap–corrected curves generated with lrm() and calibrate() from the rms package. Model goodness of fit was examined with the Hosmer–Lemeshow test using an external HL test. R function. Clinical utility was evaluated through decision curve analysis using the rmda package. A nomogram was constructed using the nomogram() function in rms and visualized interactively using the regplot package. All statistical tests were two-sided, and *p* < 0.05 indicated significance. Model performance evaluation followed a predefined validation framework assessing discrimination, calibration, and clinical usefulness across both training and validation cohorts to ensure methodological transparency and reproducibility.

## Result

### Characteristics of the training and validation sets

A total of 2,852 nurses were included, with 2,000 in the training set and 852 in the validation set. As shown in Table 1, the majority of demographic, clinical, and occupational characteristics were comparable between the two datasets, with no statistically significant differences observed in age, gender, education level, job title, hospital level, years of experience, allergy history, dermatitis history, hand hygiene training, handwashing frequency during work, hand hygiene method, disinfectant type, sanitizer use, hand-drying method, hand-care habits, glove type, and skin condition (*p* > 0.05). The only variable showing a statistically significant difference between the two cohorts was glove-wearing hours per day (*p* = 0.044), with the validation set having a slightly higher proportion of nurses reporting >2 h of glove use and the training set having more nurses reporting <0.5 h of use. All other baseline variables exhibited nonsignificant differences, indicating that the two datasets were generally well balanced and suitable for subsequent model development and validation. This slight imbalance in glove-wearing hours between the training and validation sets may have contributed modestly to differences in model performance.

**Table 1:** Baseline characteristics of the investigated nurses in the training and validation sets (*n* = 2,852).

Characteristic	Validation (*n* = 852, %)	Training (*n* = 2000, %)	*p*
OCD			0.710
No	758 (89.0)	1768 (88.4)	
Yes	94 (11.0)	232 (11.6)	
Hand_hygiene_training			0.766
No	3 (0.4)	10 (0.5)	
Yes	849 (99.6)	1990 (99.5)	
Allergy_history			0.999
No	660 (77.5)	1551 (77.6)	
Yes	192 (22.5)	449 (22.4)	
Dermatitis_history			0.454
No	696 (81.7)	1608 (80.4)	
Yes	156 (18.3)	392 (19.6)	
Gender			0.300
Male	36 (4.2)	67 (3.3)	
Female	816 (95.8)	1933 (96.7)	
Age			0.629
20–25	417 (48.9)	1004 (50.2)	
31–35	203 (23.8)	459 (23.0)	
36–40	118 (13.8)	249 (12.4)	
>40	114 (13.4)	288 (14.4)	
Hospital_level			0.280
Secondary	312 (36.6)	777 (38.9)	
Tertiary	540 (63.4)	1223 (61.2)	
Department			0.819
Internal_medicine	229 (26.9)	508 (25.4)	
Surgery	164 (19.2)	394 (19.7)	
Obstetrics_gynecology	52 (6.1)	146 (7.0)	
Pediatrics	63 (7.4)	160 (8.0)	
Emergency_critical_care	94 (11.0)	221 (11.1)	
Others	250 (29.3)	571 (28.5)	
Job_title_level			0.075
Junior	556 (65.3)	1321 (66.0)	
Intermediate	251 (29.5)	534 (26.7)	
Senior	45 (5.28)	145 (7.3)	
Education_level			0.302
Secondary_technical	23 (2.70)	36 (1.8)	
Junior_college	274 (32.2)	652 (32.6)	
Bachelor_or_above	555 (65.1)	1312 (65.6)	
Years_of_experience			0.667
<5	253 (29.7)	602 (30.1)	
6–10	273 (32.0)	634 (31.7)	
11–15	166 (19.5)	358 (17.9)	
>15	160 (18.8)	406 (20.3)	
Handwash_frequency_work			0.902
<10	30 (3.52)	66 (3.30)	
10–20	286 (33.6)	686 (34.3)	
>20	536 (62.9)	1248 (62.4)	
Hand_hygiene_method			0.429
Running_water	351 (41.2)	850 (42.5)	
Hand_disinfection	436 (51.2)	1023 (51.1)	
Surgical_disinfection	65 (7.63)	127 (6.35)	
Disinfectant_type			0.700
Alcohol	425 (49.9)	980 (49.0)	
Iodophor	260 (30.5)	652 (32.6)	
Chlorhexidine	69 (8.1)	148 (7.4)	
Others	98 (11.5)	220 (11.0)	
Sanitizer_brand			0.053
Maokang	462 (54.2)	1188 (59.4)	
3M_Avagard	94 (11.0)	209 (10.4)	
Jiarun	236 (27.7)	466 (23.3)	
Others	60 (7.04)	137 (6.85)	
Sleep_duration			1.000
<6	319 (37.4)	748 (37.4)	
>6	533 (62.6)	1252 (62.6)	
Handwash_with_soap_daily			0.306
<5	38 (4.5)	90 (4.5)	
6–10	254 (29.8)	654 (32.7)	
>10	560 (65.7)	1256 (62.8)	
Sanitizer_use_daily			0.210
<5	47 (5.5)	100 (5.1)	
6–10	217 (25.5)	573 (28.6)	
>10	588 (69.0)	1327 (66.3)	
Glove_wearing_hours_daily			0.044
<0.5	299 (35.1)	789 (39.5)	
0.5–1	201 (23.6)	494 (24.7)	
1-2	103 (12.1)	215 (10.8)	
>2	249 (29.2)	502 (25.1)	
Glove_type			0.190
Nitrile	422 (49.5)	993 (49.6)	
PE	241 (28.3)	543 (27.2)	
PVC	102 (12.0)	209 (10.4)	
Latex	87 (10.2)	255 (12.8)	
Hand_drying_method			0.485
Disposable_paper_towel	784 (92.0)	1809 (90.4)	
Automatic_dryer	21 (2.5)	54 (2.7)	
Lab_coat	20 (2.4)	67 (3.4)	
Not_dried	27 (3.1)	70 (3.5)	
Hand_cream_habit			0.720
None	231 (27.1)	538 (26.9)	
Occasionally	500 (58.7)	1177 (58.9)	
Most_of_the_time	39 (4.6)	109 (5.5)	
Always	82 (9.6)	176 (8.8)	
Hand_care_habit			0.729
None	349 (41.0)	804 (40.2)	
Occasionally	428 (50.2)	1040 (52.0)	
Most_of_the_time	31 (3.6)	68 (3.4)	
Always	44 (5.16)	88 (4.4)	
Skin_condition			0.623
Dry	259 (30.4)	647 (32.4)	
Red	20 (2.4)	38 (1.9)	
Rough	273 (32.0)	610 (30.5)	
Normal	300 (35.2)	705 (35.2)	

### Factors associated with hand OCD in nurses

Table 2 summarizes the univariate and multivariable logistic regression analyses for potential predictors of hand OCD in the training cohort. In the univariate analysis, several variables showed significant associations with OCD, including age, allergy history, dermatitis history, gender, glove type, glove-wearing hours per day, hand-cream habit, handwashing frequency at work, hospital level, job title level, skin condition, sleep duration, and years of experience (*p* < 0.05). After comparing multiple pre-specified multivariable modeling strategies during model development, a final multivariable logistic regression model was determined. In the final adjusted model, nine predictors remained independently associated with OCD. Older age was associated with increased risk (OR = 1.66, *p* < 0.001). Nurses with a history of dermatitis had substantially higher odds of developing OCD (OR = 4.43, *p* < 0.001). Occupational exposures also contributed significantly: higher-risk glove types (OR = 4.08, *p* < 0.001), longer glove-wearing duration (OR = 1.69, *p* < 0.001), and higher handwashing frequency during work (OR = 2.11, *p* < 0.001). Working in tertiary hospitals was associated with elevated risk (OR = 1.94, *p* = 0.003). Several protective factors were also identified. More frequent hand-cream use was associated with lower odds of OCD (OR = 0.68, *p* = 0.001). Better baseline skin condition reduced risk (OR = 0.77, *p* = 0.001). Adequate sleep duration was similarly protective (OR = 0.62, *p* = 0.018). Predictors such as gender, job title, and years of experience, although significant in univariate analysis, were not retained as independent predictors after adjustment.

**Table 2:** Univariate and multivariate logistic regression analysis of candidate predictors of OCD of the hands in nurses in the training set.

Variables	Univariate analysis				Multivariate analysis			
	*B*	SE	OR (95% CI)	*p*	*B*	SE	OR (95% CI)	*p*
Age	0.326	0.045	1.39 (1.27–1.51)	<0.001	0.509	0.115	1.66 (1.33–2.08)	<0.001
Allergy_history	1.735	0.146	5.67 (4.26–7.54)	<0.001				
Department	−0.048	0.035	0.95 (0.89–1.02)	0.168				
Dermatitis_history	1.904	0.148	6.71 (5.02–8.97)	<0.001	1.489	0.208	4.43 (2.95–6.66)	<0.001
Disinfectant_type	0.022	0.070	1.02 (0.89–1.17)	0.751				
Education_level	0.15	0.139	1.16 (0.88–1.53)	0.281				
Gender	−0.914	0.295	0.40 (0.22–0.71)	0.002	−0.763	0.483	0.47 (0.18–1.2)	0.114
Glove_type	1.398	0.079	4.05 (3.47–4.73)	<0.001	1.407	0.095	4.08 (3.39–4.92)	<0.001
Glove_wearing_hours_daily	0.52	0.059	1.68 (1.5–1.89)	<0.001	0.525	0.082	1.69 (1.44–1.98)	<0.001
Hand_care_habit	0.088	0.093	1.09 (0.91–1.31)	0.341				
Hand_cream_habit	−0.379	0.097	0.68 (0.57–0.83)	<0.001	−0.384	0.12	0.68 (0.54–0.86)	0.001
Hand_drying_method	0.101	0.097	1.11 (0.91–1.34)	0.300				
Hand_hygiene_method	0.106	0.116	1.11 (0.89–1.4)	0.359				
Hand_hygiene_training	13.541	460.237	759847.85 (0-Inf)	0.977				
Handwash_frequency_work	0.555	0.144	1.74 (1.31–2.31)	<0.001	0.746	0.203	2.11 (1.42–3.14)	<0.001
Handwash_with_soap_daily	0.086	0.123	1.09 (0.86–1.39)	0.487				
Hospital_level	0.549	0.154	1.73 (1.28–2.34)	<0.001	0.663	0.225	1.94 (1.25–3.02)	0.003
Job_title_level	0.252	0.105	1.29 (1.05–1.58)	0.017	−0.385	0.23	0.68 (0.43–1.07)	0.094
Sanitizer_brand	0.094	0.066	1.10 (0.97–1.25)	0.154				
Sanitizer_use_daily	−0.089	0.117	0.91 (0.73–1.15)	0.447				
Skin_condition	−0.317	0.055	0.73 (0.65–0.81)	<0.001	−0.267	0.08	0.77 (0.65–0.90)	0.001
Sleep_duration	−0.31	0.141	0.73 (0.56–0.97)	0.028	−0.485	0.205	0.62 (0.41–0.92)	0.018
Years_of_experience	0.221	0.063	1.25 (1.1–1.41)	<0.001	−0.247	0.171	0.78 (0.56–1.09)	0.147

### Discrimination performance

**Figure 1:**
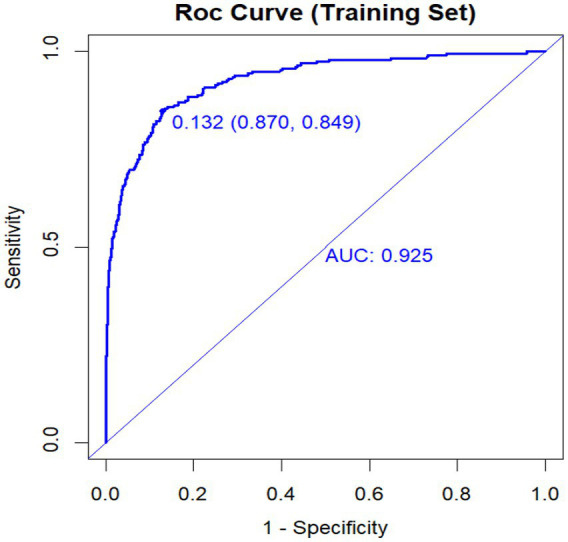
ROC curve in the training set.

**Figure 2:**
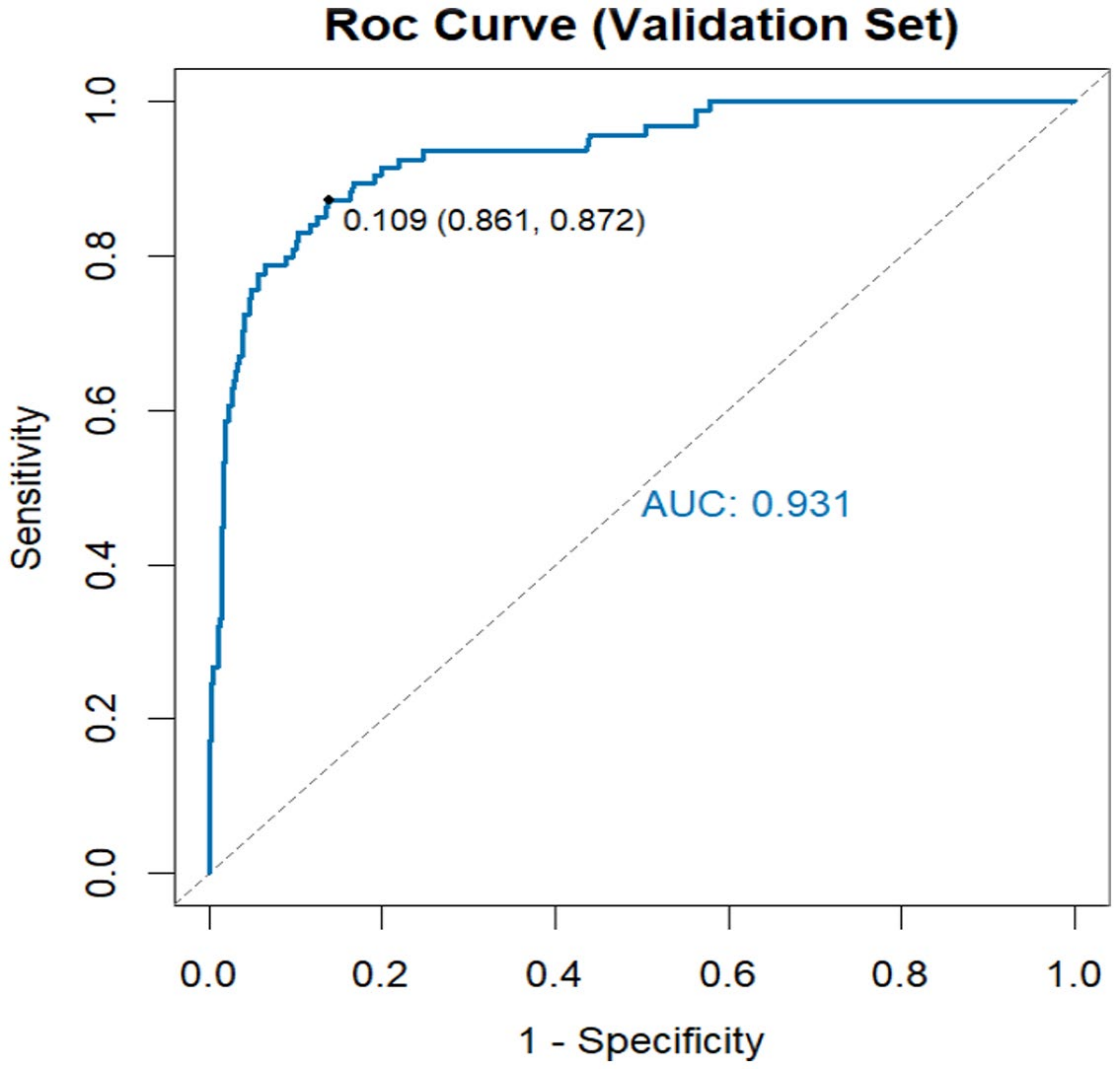
ROC curve in the validation set.

The model showed strong discriminative ability in both the training and validation datasets (Figures 1, 2). In the training cohort, the area under the ROC curve (AUC) was 0.925, with an optimal cutoff probability of 0.132, yielding a sensitivity of 0.870 and a specificity of 0.849. In the validation cohort, the AUC was 0.931, with an optimal threshold of 0.109, corresponding to a sensitivity of 0.861 and a specificity of 0.872. These findings show that the model had consistently high discrimination across both datasets. Although the AUC values were high, a certain degree of optimism or overfitting cannot be entirely excluded, even with internal split-sample validation.

Figure 1 the model had an AUC of 0.925. The optimal cutoff value was 0.132, with a sensitivity of 0.870 and a specificity of 0.849. Figure 2 the model had an AUC of 0.931. The optimal cutoff probability was 0.109, corresponding to a sensitivity of 0.861 and a specificity of 0.872.

### Calibration performance

**Figure 3:**
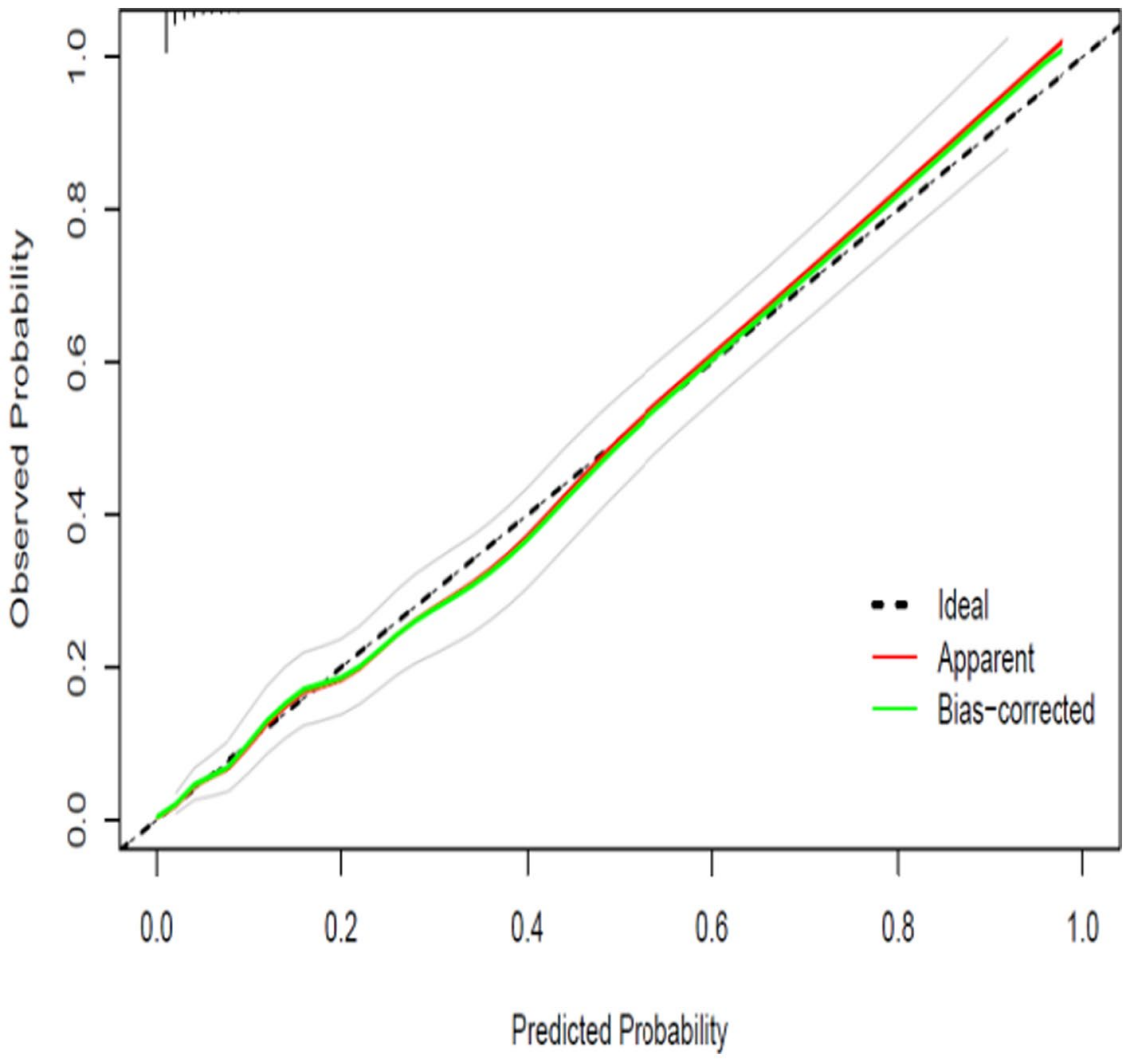
Calibration curve in the training set.

**Figure 4:**
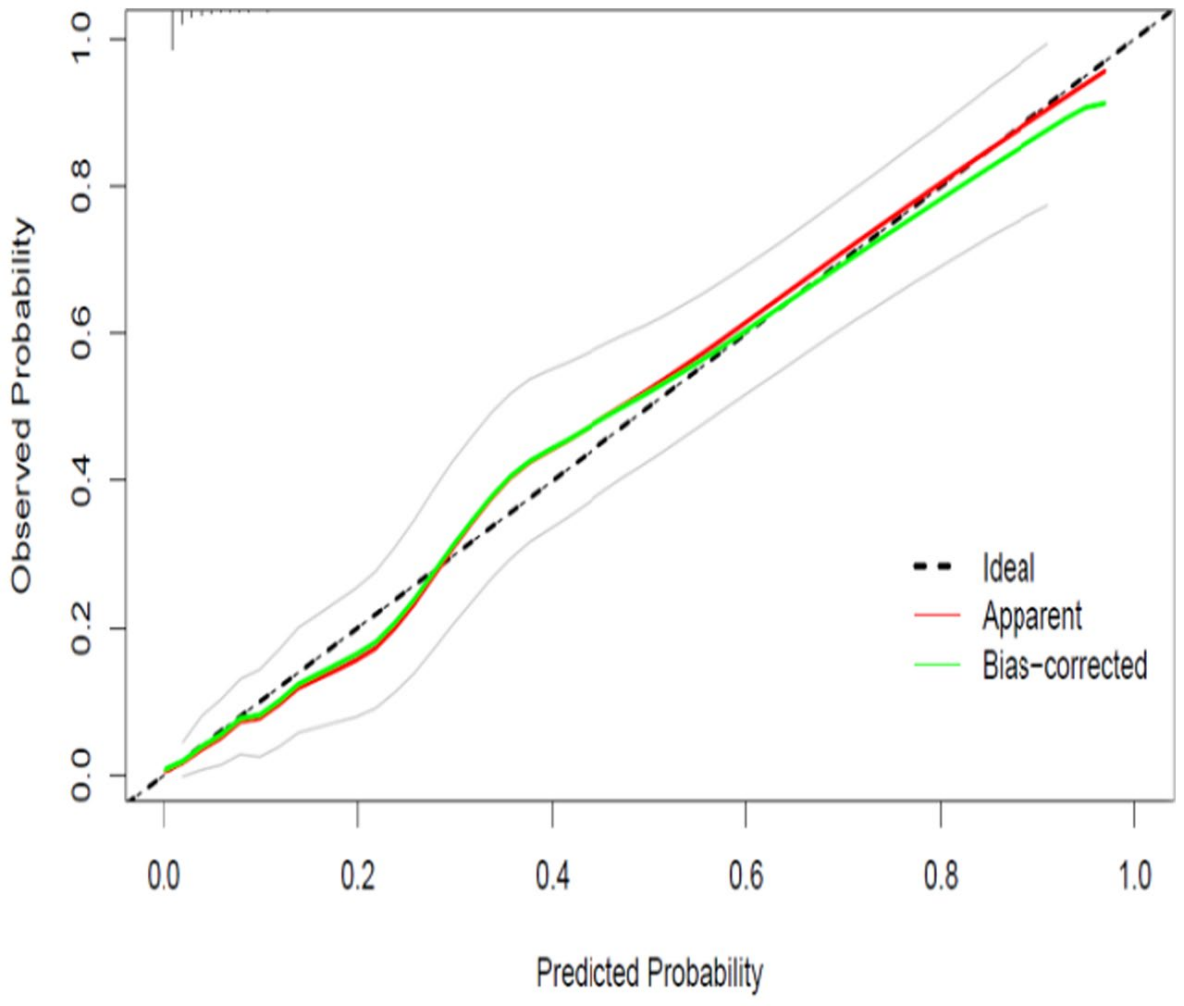
Calibration curve in the validation set.

As shown in Figures 3 and 4, the prediction model exhibited good calibration in both the training and validation cohorts. In the training set, the apparent calibration curve closely aligned with the ideal 45-degree reference line across the full range of predicted probabilities, and the bias-corrected curve based on 1,000 bootstrap resamples showed a similar pattern. A comparable trend was observed in the validation cohort, where both the apparent and bias-corrected curves demonstrated only minor deviations from the ideal reference line, indicating stable agreement between predicted and observed risks.

The calibration bands were relatively narrow in both datasets, suggesting reliable model performance across different probability levels. In addition, the extended Hosmer–Lemeshow goodness-of-fit test confirmed satisfactory calibration, with no significant lack of fit detected in either the training set (*χ*^2^ = 4.17, *p* = 0.900) or the validation set (*χ*^2^ = 9.14, *p* = 0.425). Collectively, these findings indicate that the model provides accurate absolute risk estimates across both the development and validation cohorts.

Figure 3 the plot compares predicted probabilities with observed outcomes in the training cohort. The apparent calibration curve (red line) and the bias-corrected curve after 1,000 bootstrap resamples (green line) both closely align with the ideal reference line (dashed line), indicating good calibration. Figure 4 the calibration plot in the validation cohort shows that both the apparent curve and the bootstrap bias-corrected curve maintain close agreement with the ideal 45-degree line, suggesting stable and accurate calibration.

### Clinical utility evaluation

**Figure 5:**
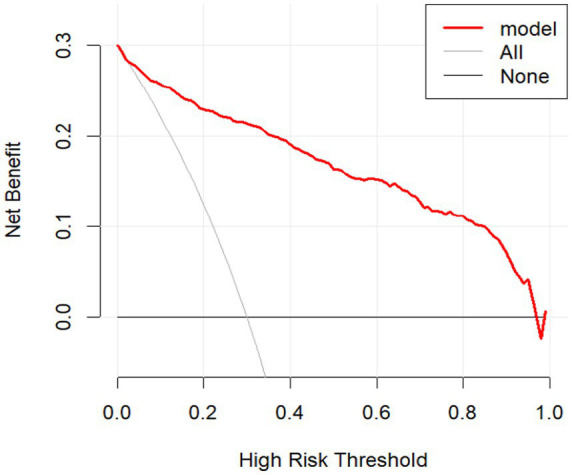
DCA curve in the training set.

**Figure 6:**
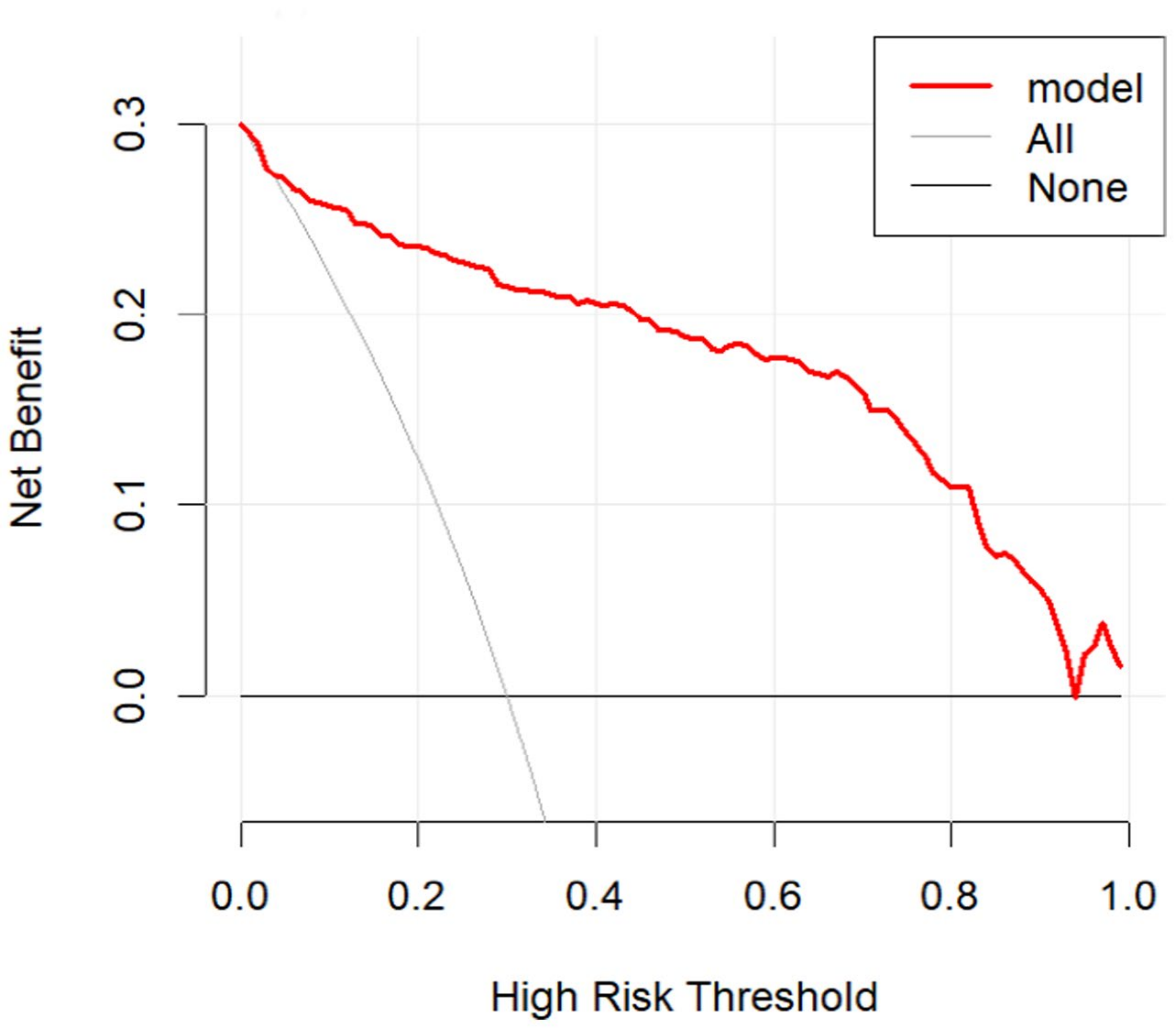
DCA curve in the validation set.

As shown in Figures 5 and 6, the decision curve analyses demonstrated that the prediction model provided a higher net benefit than both the “treat-all” and “treat-none” strategies across a broad range of threshold probabilities. In the training cohort, the model achieved consistently superior net benefit from 0.01 to 0.98, indicating stable performance across nearly the full threshold range. A similar pattern was observed in the validation cohort, where the model continued to outperform the reference strategies across threshold probabilities from 0.02 to 0.96. These findings indicate that the model maintains consistent clinical utility in both the training and validation datasets.

### Nomogram construction

**Figure 7:**
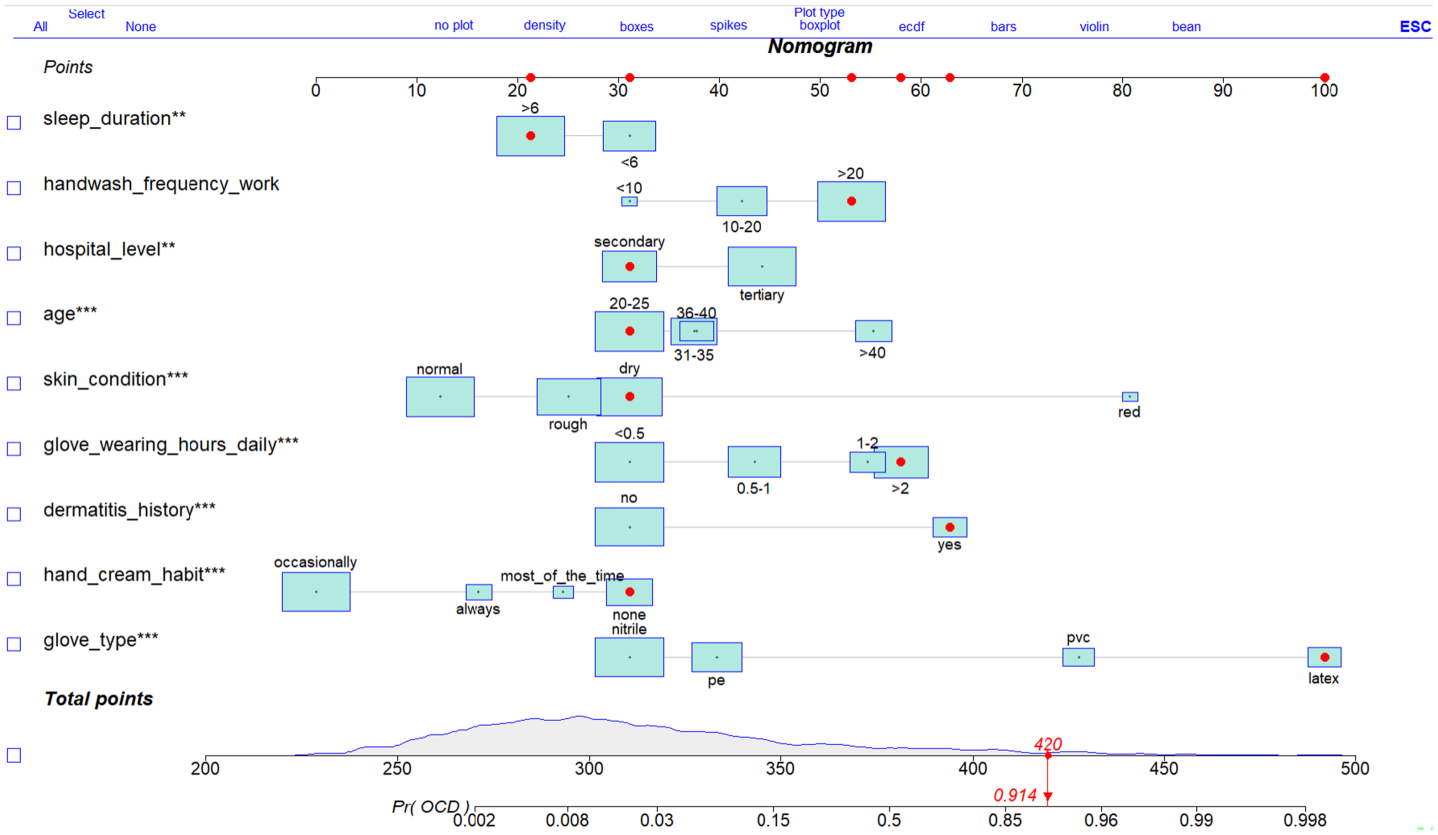
Nomogram for predicting hand OCD among nurses.

As shown in Figure 7, a nomogram was developed based on the final multivariable logistic regression model to enable individualized prediction of occupational contact dermatitis among nurses. The nomogram integrates nine significant predictors—sleep duration, handwashing frequency during work, hospital level, age, skin condition, glove-wearing hours per day, dermatitis history, hand-cream use frequency, and glove type—each assigned a corresponding point value according to its relative contribution to the model. By summing the points for all applicable predictor categories, a total score is generated, which maps directly to an estimated probability of developing occupational contact dermatitis. The graphical layout provides a practical tool for visualizing and quantifying risk at the individual level within the study population.

Figure 7 the nomogram integrates nine independent predictors from the final multivariable logistic regression model, including sleep duration, handwashing frequency during work, hospital level, age, skin condition, glove-wearing hours per day, dermatitis history, hand-cream use frequency, and glove type. Total points correspond to an individualized predicted probability of developing occupational contact dermatitis.

## Discussion

In this multicenter study of 2,852 nurses from 40 hospitals across multiple Chinese provinces, we developed and internally validated a clinical prediction model for OCD of the hands. The final model included nine independent predictors—age, dermatitis history, glove type, glove-wearing duration, hand-cream use frequency, handwashing frequency at work, hospital level, baseline skin condition, and sleep duration—selected through a structured multivariable modeling process. The model demonstrated high discrimination, with area under the curve (AUC) values of 0.925 in the training cohort and 0.931 in the validation cohort. Calibration was excellent in both datasets, as predicted risks closely matched observed outcomes and Hosmer–Lemeshow tests indicated good model fit. Decision curve analysis (DCA) further showed that the model yielded a greater net benefit across a wide range of threshold probabilities compared to “treat-all” or “treat-none” approaches. Together, these findings—coupled with the development of an easy-to-use nomogram for individualized risk estimation—indicate that our model provides an accurate and clinically useful tool for predicting OCD risk among nurses.

### Comparison with previous studies

Overall, our results align with existing research on occupational skin disease in healthcare workers, while also highlighting some unique patterns. Consistent with earlier reports, a history of dermatitis emerged as one of the strongest risk factors for OCD, underscoring that prior skin barrier damage markedly increases susceptibility to irritant or allergic reactions (15). Glove-related exposures—specifically the type of glove material and prolonged daily glove use—were also strongly associated with OCD, aligning with evidence that occlusion, sweating, and allergens from latex or rubber additives contribute significantly to hand dermatitis in nurses (16, 17). Frequent handwashing at work remained a robust predictor, supporting reports that repeated use of soaps and disinfectants can disrupt the skin’s protective barrier (18). Conversely, we found that regular use of hand cream was protective, consistent with findings that skin-care intervention programs can mitigate the risk of occupational dermatitis (19).

Notably, our model identified two predictors rarely considered in prior studies: sleep duration and hospital level. This novel finding suggests that broader occupational and organizational factors may influence OCD risk beyond direct chemical exposures. For instance, insufficient sleep could impair skin barrier recovery and immune function (20, 21), while working in a higher-tier hospital may indicate greater workload or stricter hygiene protocols that elevate dermatitis risk. However, given the cross-sectional design of the present study, these associations should be interpreted with caution, and causal or mechanistic inferences cannot be established. Finally, the overall prevalence of hand OCD in our cohort (~11%) was comparable to international reports (22, 23), indicating that our multicenter Chinese sample reflects global patterns of occupational dermatitis among nurses.

### Methodological strengths

An important strength of our study is the rigorous, state-of-the-art approach to model development. We adhered to contemporary clinical prediction modeling guidelines (e.g., TRIPOD) (14) by pre-specifying candidate predictors, testing multiple multivariable modeling strategies, and incorporating a predefined training/validation split for model evaluation. In the modeling stage, we explored four different logistic regression variable-selection methods (enter, forward, backward, and stepwise), comparing their performance using Akaike’s Information Criterion and likelihood ratio tests (24, 25). This systematic process ensured that the final model achieved an optimal balance between simplicity and predictive accuracy. Although external validation was not performed, the multicenter design and internal split-sample validation provided an initial assessment of model generalizability. Future prospective studies with independent cohorts are warranted for external validation.

### Model performance and utility

The final model exhibited excellent performance and demonstrated generalizability within our dataset. Moreover, decision curve analysis supported the model’s potential clinical utility. Finally, we translated the model into a user-friendly nomogram, which offers clinicians a simple visual tool to estimate an individual nurse’s OCD risk based on multiple factors. This nomogram can facilitate early risk stratification and personalized preventive counseling in routine occupational health practice. Nevertheless, although the model demonstrated excellent discrimination and calibration using internal split-sample validation, a certain degree of model optimism or overfitting cannot be entirely excluded, particularly when applied beyond the original study setting. Internal validation primarily reflects reproducibility within the same underlying population and does not fully guarantee performance in independent settings. Therefore, external validation in geographically and institutionally distinct nurse populations is warranted to further confirm the model’s robustness and real-world transportability. Differences in healthcare systems, occupational health policies, and infection-control practices across countries may necessitate recalibration or contextual adaptation before broader international application.

### Limitations

Despite its strengths, this study has several limitations that should be acknowledged: Cross-sectional design: Because the study is cross-sectional, we cannot infer causality, and there is potential temporal ambiguity between exposures and outcomes (26). Specifically, exposures and outcomes were assessed at the same time, which limits causal interpretation of the observed associations. This lack of temporal sequencing may also influence the stability of estimated associations, making it difficult to distinguish transient correlations from sustained risk factors that meaningfully contribute to long-term risk estimation. Self-reported data: All information was obtained via a self-administered online questionnaire. This may introduce recall bias, exposure misclassification, or inconsistent reporting (27). Although OCD outcomes were based on participants’ reports of clinician-confirmed diagnoses, most exposure variables relied on self-report, which may affect the accuracy of risk estimation. Misclassification of self-reported exposure variables may have led to attenuation or inflation of estimated effect sizes, thereby influencing individual risk estimates generated by the model. In particular, non-differential misclassification would be expected to bias associations toward the null, whereas differential reporting related to symptom awareness could result in overestimation of risk in certain subgroups. Outcome assessment: The diagnosis of OCD was based on participants’ reports of clinician-confirmed episodes without a contemporaneous dermatological examination, which could affect diagnostic accuracy. Sample representativeness: As the study population was drawn exclusively from Chinese hospitals, certain subgroups of nurses (e.g., male nurses, those using specific disinfectant brands, or surgical staff) were underrepresented in our sample, potentially limiting the model’s generalizability to other healthcare systems or those populations. Validation scope: We validated the model using an internal split-sample approach. While this adheres to TRIPOD recommendations, internal validation alone cannot fully exclude potential optimism or overfitting. Therefore, independent external validation using data from other institutions or regions is needed to further confirm the model’s robustness and real-world transportability. In addition, model recalibration or adaptation may be required when applying the tool in other healthcare systems or countries with different work practices, glove materials, and infection-control protocols.

### Future directions

To further improve the model and enhance its clinical applicability, future research should consider several avenues: Conducting prospective cohort studies to establish clear temporal relationships between exposure factors and the development of OCD. Incorporating objective clinical assessments (such as dermatologist-confirmed evaluations or skin barrier measurements) to validate outcomes and reduce misclassification. Integrating environmental or biochemical exposure data (e.g., quantification of common allergens or analysis of glove material composition) for a more comprehensive risk assessment. Performing external validation studies in different regions or healthcare systems to test the model’s generalizability across diverse nurse populations. Developing digital risk-assessment tools (for example, integrating the nomogram into hospital infection-control software) to enable real-time monitoring and personalized prevention strategies for occupational dermatitis. Implementing these steps could refine the model’s predictive accuracy, support its broader implementation in occupational health practice, and deepen our understanding of factors influencing nurses’ skin health.

## Conclusion

In this multicenter cross-sectional study involving 2,852 nurses from 40 hospitals, we established and validated a clinical prediction model for OCD of the hands using routinely obtainable demographic, occupational, and behavioral factors. The final model, incorporating nine independent predictors—including age, dermatitis history, glove type, glove-wearing duration, handwashing frequency at work, hand cream habit, hospital level, baseline skin condition, and sleep duration—showed strong discriminative ability and good calibration in both development and validation cohorts. The corresponding nomogram provides an intuitive tool for estimating individualized OCD risk and may support early identification of high-risk nurses and targeted preventive strategies in clinical settings. While the cross-sectional design limits causal inference and the reliance on self-reported exposures and clinically diagnosed outcomes may introduce minor reporting bias, the model demonstrates robust performance and practical utility across diverse hospital environments. Future studies should consider prospective validation, exploration of additional environmental or biochemical predictors, and integration of the model into digital platforms to enhance occupational skin-disease surveillance and guide personalized preventive interventions for the nursing workforce.

## Data Availability

The original contributions presented in the study are included in the article/Supplementary material, further inquiries can be directed to the corresponding authors.
