# Supplementary material for: Occupational skin health risk among nurses: development and validation of a nomogram-based prediction model
Source: Front Public Health. 2026 Mar 31;14:1748717. doi: 10.3389/fpubh.2026.1748717 (PMC13078389; doi:10.3389/fpubh.2026.1748717)
Supplement: Supplementary file 2 [file Table_1.docx]

Set the working path

getwd()

setwd("D:/R work")

#变量理解

#OCD 0=no 1=yes

#dataset 0=vad 1=dev

#gender 1 = male 2 = female

#age 1 = 20–25 2 = 26–30 3 = 31–35 4 = 36–40 5 = >40

#hospital_level 1 = secondary 2 = tertiary

#department 1 = internal_medicine 2 = surgery 3 = obstetrics_gynecology 4 = pediatrics 5 = emergency_critical_care 6 = others

#job_title_level 1 = junior 2 = intermediate 3 = senior

#education_level 1 = secondary_technical 2 = junior_college 3 = bachelor_or_above

#years_of_experience 1 = <5 2 = 6–10 3 = 11–15 4 = >15

#allergy_history 0 = no 1 = yes

#dermatitis_history 0 = no 1 = yes

#handwash_frequency_work 1 = <10 2 = 10–20 3 = >20

#hand_hygiene_method 1 = running_water 2 = hand_disinfection 3 = surgical_disinfection

#disinfectant_type 1 = alcohol 2 = iodophor 3 = chlorhexidine 4 = others

#sanitizer_brand 1 = Maokang 2 = 3M_Avagard 3 = Jiarun 4 = others

#sleep_duration 1 = <6h 2 = >6h

#handwash_with_soap_daily 1 = <5 2 = 6–10 3 = >10

#sanitizer_use_daily 1 = <5 2 = 6–10 3 = >10

#glove_wearing_hours_daily 1 = <0.5h 2 = 0.5–1h 3 = >1–2h 4 = >2h

#glove_type 1=nitrile 2=pe 3=pvc 4=latex

#hand_hygiene_training 0 = no 1 = yes

#hand_drying_method 1 = disposable_paper_towel 2 = automatic_dryer 3 = lab_coat 4 = not_dried

#hand_cream_habit 1 = none 2 = occasionally 3 = most_of_the_time 4 = always

#hand_care_habit 1 = none 2 = occasionally 3 = most_of_the_time 4 = always

#skin_condition 1 = dry 2 = red 3 = rough 4 = normal

install.packages("readr")

library(readr)

mydata <- read_csv("OCD data.csv")

mydata<-na.omit(mydata)

View(mydata)

names(mydata)

str(mydata)

attach(mydata)

install.packages("caret")

library(caret)

set.seed(123)

trianandvad<- createDataPartition(y=mydata$ID,p=0.70,list=FALSE)

train <- mydata[trianandvad, ]

vadi<-mydata[-trianandvad,]

View(train)

View(vadi)

write.csv(train, "train.csv")

write.csv(vadi, "vadi.csv")

dev = mydata[mydata$dataset==1,]

vad = mydata[mydata$dataset==0,]

View(dev)

View(vad)

write.csv(dev, "dev.csv")

write.csv(vad, "vad.csv")

install.packages("compareGroups")

library(compareGroups)

library(glue)

library(tidyr)

library(broom)

mydata$OCD <-

factor(mydata$OCD,

levels = c(0,1),

labels = c("no", "yes"))

mydata$dataset <-

factor(mydata$dataset,

levels = c(0,1),

labels = c("Vad", "dev"))

mydata$gender <-

factor(mydata$gender,

levels = c(1,2),

labels = c("male", "female"))

mydata$age <-

factor(mydata$age,

levels = c(1,2,3,4,5),

labels = c("20-25", "26-30","31-35", "36-40",">40"))

mydata$hospital_level <-

factor(mydata$hospital_level,

levels = c(1,2),

labels = c("secondary", "tertiary"))

mydata$department <-

factor(mydata$department,

levels = c(1,2,3,4,5,6),

labels = c("internal_medicine", "surgery","obstetrics_gynecology", "pediatrics","emergency_critical_care","others"))

mydata$job_title_level <-

factor(mydata$job_title_level,

levels = c(1,2,3),

labels = c("junior", "intermediate","senior"))

mydata$education_level <-

factor(mydata$education_level,

levels = c(1,2,3),

labels = c("secondary_technical", "junior_college","bachelor_or_above"))

mydata$years_of_experience <-

factor(mydata$years_of_experience,

levels = c(1,2,3,4),

labels = c("<5", "6-10","11-15",">15"))

mydata$allergy_history <-

factor(mydata$allergy_history,

levels = c(0,1),

labels = c("no", "yes"))

mydata$dermatitis_history <-

factor(mydata$dermatitis_history,

levels = c(0,1),

labels = c("no", "yes"))

mydata$handwash_frequency_work <-

factor(mydata$handwash_frequency_work,

levels = c(1,2,3),

labels = c("<10", "10-20",">20"))

mydata$hand_hygiene_method <-

factor(mydata$hand_hygiene_method,

levels = c(1,2,3,4),

labels = c("running_water", "hand_disinfection","surgical_disinfection","others"))

mydata$disinfectant_type <-

factor(mydata$disinfectant_type,

levels = c(1,2,3,4),

labels = c("alcohol", "iodophor","chlorhexidine","others"))

mydata$sanitizer_brand <-

factor(mydata$sanitizer_brand,

levels = c(1,2,3,4),

labels = c("Maokang", "3M_Avagard","Jiarun","others"))

mydata$sleep_duration <-

factor(mydata$sleep_duration,

levels = c(1,2),

labels = c("<6", ">6"))

mydata$handwash_with_soap_daily <-

factor(mydata$handwash_with_soap_daily,

levels = c(1,2,3),

labels = c("<5", "6-10",">10"))

mydata$sanitizer_use_daily <-

factor(mydata$sanitizer_use_daily,

levels = c(1,2,3),

labels = c("<5", "6-10",">10"))

mydata$glove_wearing_hours_daily <-

factor(mydata$glove_wearing_hours_daily,

levels = c(1,2,3,4),

labels = c("<0.5", "0.5-1","1-2",">2"))

mydata$glove_type <-

factor(mydata$glove_type,

levels = c(1,2,3,4),

labels = c("nitrile", "pe","pvc","latex"))

mydata$hand_hygiene_training <-

factor(mydata$hand_hygiene_training,

levels = c(0,1),

labels = c("no", "yes"))

mydata$hand_drying_method <-

factor(mydata$hand_drying_method,

levels = c(1,2,3,4),

labels = c("disposable_paper_towel", "automatic_dryer","lab_coat","not_dried"))

mydata$hand_cream_habit <-

factor(mydata$hand_cream_habit,

levels = c(1,2,3,4),

labels = c("none", "occasionally","most_of_the_time","always"))

mydata$hand_care_habit <-

factor(mydata$hand_care_habit ,

levels = c(1,2,3,4),

labels = c("none", "occasionally","most_of_the_time","always"))

mydata$skin_condition <-

factor(mydata$skin_condition ,

levels = c(1,2,3,4),

labels = c("dry", "red","rough","normal"))

str(mydata)

table1<-descrTable(dataset ~ ., data = mydata,show.all = TRUE)

table1<-descrTable(dataset ~ ., data = mydata)

print(table1)

export2csv(table1,file = "table1.csv")

uni_glm_model<-function(x){

FML<-as.formula(paste0("OCD==1~",x))

glm1<-glm(FML,data = dev,family = binomial)

glm2<-summary(glm1)

OR<-round(exp(coef(glm1)),2)

SE<-round(glm2$coefficients[,2],3)

CI2.5<-round(exp(coef(glm1)-1.96*SE),2)

CI97.5<-round(exp(coef(glm1)+1.96*SE),2)

CI<-paste0(CI2.5,'-',CI97.5)

B<-round(glm2$coefficients[,1],3)

Z<-round(glm2$coefficients[,3],3)

P<-round(glm2$coefficients[,4],3)

uni_glm_model<-data.frame('characteristics'=x,

'B'=B,

'SE'=SE,

'OR'=OR,

'CI'=CI,

'Z' =Z,

'P'=P)[-1,]

uni_glm_model$characteristics=rownames(uni_glm_model)

return(uni_glm_model)

}

variable.names<-colnames(dev)[c(4:26)]

variable.names

uni_glm<-lapply(variable.names,uni_glm_model)

uni_glm

install.packages("plyr")

library(plyr)

uni_glm<-ldply(uni_glm,data.frame)

uni_glm

View(uni_glm)

write.csv(uni_glm, "uni.csv")

uni_glm1 <- uni_glm[uni_glm$P<= 0.05,]

uni_glm1

uni_glm$characteristics[uni_glm$P<= 0.05]

write.csv(uni_glm1, "p5.csv")

fml<- as.formula(paste0('OCD==1~',paste0(uni_glm$characteristics[uni_glm$P<0.05],collapse = '+')))

fml

fml<-as.formula(OCD == 1 ~ allergy_history + dermatitis_history + gender +

age + hospital_level + job_title_level + years_of_experience + handwash_frequency_work + sleep_duration + glove_wearing_hours_daily + glove_type + hand_cream_habit + skin_condition)

fml

modelA<-glm(fml,data = dev,family=binomial)

modelA

summary(modelA)

#2.—forward

modelX<-glm(OCD==1~1,data = dev,family=binomial)

modelX

#modelB

modelB<-step(modelX,scope=list(upper=~ allergy_history + dermatitis_history + gender +

age + hospital_level + job_title_level + years_of_experience +

handwash_frequency_work + sleep_duration + glove_wearing_hours_daily +

glove_type + hand_cream_habit + skin_condition,

lower=~1),data = dev,family=binomial,direction ="forward")

summary(modelB)

#3.-backward

modelC<-step(modelA,direction ="backward")

summary(modelC)

#4.-both

modelD<-step(modelA,direction = "both")

summary(modelD)

#95%CI

cbind(coef=coef(modelD),confint(modelD))

#OR及95%CI

exp(cbind(OR=coef(modelD),confint(modelD)))

#AIC

AIC(modelA,modelB,modelC,modelD)

#AIC

anova(modelA,modelC,test = "Chisq")

anova(modelA,modelD,test = "Chisq")

anova(modelA,modelB,test = "Chisq")

anova(modelB,modelD,test = "Chisq")

anova(modelC,modelB,test = "Chisq")

anova(modelC,modelD,test = "Chisq")

#modelD result

modelD<-step(modelA,direction = "both")

modelD

glm3<-summary(modelD)

glm3

glm3$coefficients

OR<-round(exp(glm3$coefficients[,1]),2)

OR

SE<-round(glm3$coefficients[,2],3)

CI2.5<-round(exp(coef(modelD)-1.96*SE),2)

CI97.5<-round(exp(coef(modelD)+1.96*SE),2)

CI<-paste0(CI2.5,'-',CI97.5)

#CI

B<-round(glm3$coefficients[,1],3)

#Z<-round(glm3$coefficients[,3],3)

P<-round(glm3$coefficients[,4],3)

##'characteristics'=multiname,

mlogit<-data.frame(

'B'=B,

'SE'=SE,

'OR'=OR,

'CI'=CI,

'Z' =Z,

'P'=P)[-1,]

mlogit

characteristics=rownames(mlogit)

characteristics

mlogit<-cbind(characteristics,mlogit)

mlogit

mlogit<-mlogit[-1,]

View(mlogit)

write.csv(mlogit, "multi.csv")

table2<-merge.data.frame(uni_glm,mlogit,by='characteristics',all = TRUE,sort = T)

table2

#View(table2)

write.csv(final, "table2.csv")

getwd()

setwd("D:/R work")

library(readr)

mydata <- read_csv("OCD data.csv")

dev = mydata[mydata$dataset==1,]

vad = mydata[mydata$dataset==0,]

#View(dev)

#View(vad)

fml9<-as.formula(OCD == 1 ~ age + dermatitis_history + glove_type +

glove_wearing_hours_daily + hand_cream_habit + handwash_frequency_work + hospital_level + skin_condition + sleep_duration )

model9<-glm(fml9,data = dev,family = binomial(logit))

dev$predmodel9<- predict(newdata=dev,model9,"response")

#View(dev)

vad$predmodel9<- predict(newdata=vad,model9,"response")

#View(vad)

install.packages("pROC")

library(pROC)

devmodelA <- roc(OCD~predmodel9, data = dev,smooth=F)

#devmodelA <- roc(hypoglycemia~predmodel9, data = dev,smooth=T) #如果做平滑，建议不做

devmodelA

round(auc(devmodelA),3)

round(ci(auc(devmodelA)),3)

plot(devmodelA,

print.auc=TRUE,

print.thres=TRUE,

legacy.axes=TRUE,

main = "Roc Curve (Training Set)",

col= "blue",

print.thres.col="blue",

identity.col="blue",

identity.lty=1,

identity.lwd=1)

vadmodelA <- roc(OCD~predmodel9, data = vad,smooth=F)

round(auc(vadmodelA),3)

round(ci(auc(vadmodelA)),3)

#ROC1

plot(vadmodelA,

main = "Roc Curve (Validation Set)",

col = "#0072B2", lwd = 2,

legacy.axes = TRUE, # Specificity

xlim = c(1,0), ylim = c(0,1),

print.auc = TRUE,

print.thres = TRUE, print.thres.cex = 0.8,

identity.col = "gray40", identity.lty = 2, identity.lwd = 1.2)

getwd()

setwd("D:/R work")

library(readr)

mydata <- read_csv("OCD data.csv")

mydata<-na.omit(mydata)

attach(mydata)

dev = mydata[mydata$dataset==1,]

vad = mydata[mydata$dataset==0,]

fml9<-as.formula(OCD == 1 ~ age + dermatitis_history + glove_type +

glove_wearing_hours_daily + hand_cream_habit + handwash_frequency_work + hospital_level + skin_condition + sleep_duration )

model9<-glm(fml9,data = dev,family = binomial(logit))

dev$predmodel9<- predict(newdata=dev,model9,"response")

#View(dev)

vad$predmodel9<- predict(newdata=vad,model9,"response")

install.packages("calibrate")

library(calibrate)

library(MASS)

install.packages("rms")

library(rms)

val.prob(dev$predmodel9,dev$OCD)

val.prob(vad$predmodel9,vad$OCD)

source("HLtest.R") #一定要把HLtest.R放在之前设定的起始目录中。

hl.ext2(dev$predmodel9,dev$OCD)

source("HLtest.R") #一定要把HLtest.R放在之前设定的起始目录中。

hl.ext2(vad$predmodel9,vad$OCD)

install.packages("rms")

library(rms)

fml<-OCD==1 ~age + dermatitis_history + glove_type +

glove_wearing_hours_daily + hand_cream_habit + handwash_frequency_work + hospital_level + skin_condition + sleep_duration

fit3<-lrm(fml,data=dev,x=TRUE,y=TRUE)

cal3<-calibrate(fit3,method="boot",B=1000) #Bootstrap

plot(cal3,

xlim = c(0,1),

xlab = "Predicted Probability",

ylab = "Observed Probability",

legend=FALSE,

subtitles = FALSE)

abline(0,1,col="black",lty=2,lwd=2)

lines(cal3[,c("predy","calibrated.orig")],type = "l",lwd=2,col="red",pch=16)

lines(cal3[,c("predy","calibrated.corrected")],type = "l",lwd=2,col="green",pch=16)

legend(0.75,0.35,

c("Ideal","Apparent","Bias-corrected"),

lty = c(2,1,1),

lwd = c(2,1,1),

col = c("black","red","green"),

bty = "n")

fit4<-lrm(fit3,data=vad,x=TRUE,y=TRUE)

cal4<-calibrate(fit4,method="boot",B=1000)

plot(cal4,

xlim = c(0,1),

xlab = "Predicted Probability",

ylab = "Observed Probability",

legend=FALSE,

subtitles = FALSE)

abline(0,1,col="black",lty=2,lwd=2)

lines(cal4[,c("predy","calibrated.orig")],type = "l",lwd=2,col="red",pch=16)

lines(cal4[,c("predy","calibrated.corrected")],type = "l",lwd=2,col="green",pch=16)

legend(0.75,0.35,

c("Ideal","Apparent","Bias-corrected"),

lty = c(2,1,1),

lwd = c(2,1,1),

col = c("black","red","green"),

bty = "n")

install.packages("rmda")

library(rmda)

model<-decision_curve(OCD ~ age + dermatitis_history + glove_type +

glove_wearing_hours_daily + hand_cream_habit + handwash_frequency_work + hospital_level + skin_condition + sleep_duration,

data = dev,

family = binomial(logit),

thresholds = seq(0,1,by=0.01),

confidence.intervals = 0.95,

study.design = 'case-control',

population.prevalence =0.3)

plot_decision_curve(model,curve.names = c('model'),

xlim = c(0,1.0),

cost.benefit.axis = F,

col = c('red'),

confidence.intervals = F,

standardize = F)

fml9<-as.formula(OCD == 1 ~ age + dermatitis_history + glove_type +

glove_wearing_hours_daily + hand_cream_habit + handwash_frequency_work + hospital_level + skin_condition + sleep_duration)

model9<-glm(fml9,data = vad,family = binomial(logit))

vad$predmodel9<- predict(newdata=vad,model9,"response")

model<-decision_curve(OCD ~ age + dermatitis_history + glove_type +

glove_wearing_hours_daily + hand_cream_habit + handwash_frequency_work + hospital_level + skin_condition + sleep_duration,

data = vad,

family = binomial(logit),

thresholds = seq(0,1,by=0.01),

confidence.intervals = 0.95,

study.design = 'case-control',

population.prevalence =0.3)

plot_decision_curve(model,curve.names = c('model'),

xlim = c(0,1.0),

cost.benefit.axis = F,

col = c('red'),

confidence.intervals = F,

standardize = F)

library(readr)

mydata <- read_csv("OCD data.csv")

mydata<-na.omit(mydata)

str(mydata)

mydata$OCD <-

factor(mydata$OCD,

levels = c(0,1),

labels = c("no", "yes"))

mydata$age <-

factor(mydata$age,

levels = c(1,2,3,4,5),

labels = c("20-25", "26-30","31-35", "36-40",">40"))

mydata$hospital_level <-

factor(mydata$hospital_level,

levels = c(1,2),

labels = c("secondary", "tertiary"))

mydata$dermatitis_history <-

factor(mydata$dermatitis_history,

levels = c(0,1),

labels = c("no", "yes"))

mydata$handwash_frequency_work <-

factor(mydata$handwash_frequency_work,

levels = c(1,2,3),

labels = c("<10", "10-20",">20"))

mydata$sleep_duration <-

factor(mydata$sleep_duration,

levels = c(1,2),

labels = c("<6", ">6"))

mydata$glove_wearing_hours_daily <-

factor(mydata$glove_wearing_hours_daily,

levels = c(1,2,3,4),

labels = c("<0.5", "0.5-1","1-2",">2"))

mydata$glove_type <-

factor(mydata$glove_type,

levels = c(1,2,3,4),

labels = c("nitrile", "pe","pvc","latex"))

mydata$hand_cream_habit <-

factor(mydata$hand_cream_habit,

levels = c(1,2,3,4),

labels = c("none", "occasionally","most_of_the_time","always"))

mydata$skin_condition <-

factor(mydata$skin_condition ,

levels = c(1,2,3,4),

labels = c("dry", "red","rough","normal"))

dev = mydata[mydata$dataset==1,]

vad = mydata[mydata$dataset==0,]

library(rms)

ddist <- datadist(dev)

options(datadist='ddist')

model <- lrm(OCD ~age + dermatitis_history + glove_type +

glove_wearing_hours_daily + hand_cream_habit + handwash_frequency_work + hospital_level + skin_condition + sleep_duration,data=dev)

nomomodel <- nomogram(model,lp=F,

fun=function(x)1/(1+exp(-x)),

fun.at=seq(0.1,1,by=0.1),

funlabel="Diagnostic possibility")

nomomodel

plot(nomomodel)

install.packages("regplot")

library(regplot)

model <- glm(OCD ~age + dermatitis_history + glove_type +

glove_wearing_hours_daily + hand_cream_habit + handwash_frequency_work + hospital_level + skin_condition + sleep_duration, data = dev, family = binomial(link="logit"))

regplot(model,observation=dev[100,])

regplot(model,

observation=dev[3,],

center=TRUE,

title = "Nomogram",

points = TRUE,

odds = FALSE,

showP = TRUE,

rank = "sd",

clickable = TRUE)
